# Monocryl^®^ vs. Monocryl Plus^®^ in Pediatric Reconstructive Urological Surgery: Outcomes of 653 Patients over 18 Years at a Single Centre

**DOI:** 10.3390/medsci14010099

**Published:** 2026-02-19

**Authors:** Zenon Pogorelić, Ivan Lovrinčević, Jakov Todorić, Dražen Budimir, Jasenka Kraljević

**Affiliations:** 1 Department of Pediatric Surgery, University Hospital of Split, 21000 Split, Croatia; 2 Department of Surgery, School of Medicine, University of Split, 21000 Split, Croatia; 3 Department of Surgery, University Hospital of Split, 21000 Split, Croatia

**Keywords:** pediatric urology, hypospadias, vesicoureteral reflux, hydronephrosis, surgical site infection, SSI, antibacterial-coated sutures, monocryl plus

## Abstract

Background: Surgical site infection (SSI) remains a major concern in pediatric urological reconstructive surgery. Antibacterial-coated absorbable sutures like Monocryl Plus^®^ have been introduced to reduce SSI, but evidence in pediatric populations is limited. This study aimed to compare outcomes between Monocryl^®^ and Monocryl Plus^®^ sutures in common pediatric urological procedures. Methods: A retrospective review was conducted of all children who underwent reconstructive urological surgery for hydronephrosis, vesicoureteral reflux (VUR), or hypospadias at the University Hospital of Split between January 2008 and December 2025. A total of 653 patients were included: 149 with hydronephrosis, 187 with VUR, and 317 with hypospadias. Patients were grouped based on suture type (Monocryl^®^ vs. Monocryl Plus^®^). The primary outcome was SSI within 30 days after surgery; secondary outcomes included overall complications, reoperations, readmissions, and length of hospital stay. Results: SSI occurred less frequently with Monocryl Plus than with Monocryl (3.8% vs. 6.9%, *p* = 0.04). The median length of stay was shorter in the Monocryl Plus group (5 days, IQR 4–7) compared to Monocryl (6 days, IQR 5–8; *p* = 0.02). Overall complication rates were 6.1% vs. 10.0% (*p* = 0.07), early complications 4.6% vs. 8.0% (*p* = 0.06), and late complications 2.3% vs. 4.2% (*p* = 0.18), favouring Monocryl Plus but without statistical significance. Reoperation was required in 1.8% vs. 3.4% (*p* = 0.19), and readmission in 2.6% vs. 5.0% (*p* = 0.12). Subgroup analysis showed minimal differences in hydronephrosis (all *p* > 0.6), modest reductions in VUR (SSI 8.1% vs. 4.4%, *p* = 0.21), and significant differences in hypospadias (SSI 7.8% vs. 4.2%, *p* = 0.04; fistula 12.2% vs. 6.5%, *p* = 0.03). Multivariate regression confirmed Monocryl Plus as independently associated with lower odds of SSI (OR 0.55, 95% CI 0.30–0.98, *p* = 0.04) and prolonged hospitalization >7 days (OR 0.59, 95% CI 0.38–0.91, *p* = 0.02). Conclusions: In pediatric urological reconstructive surgery, Monocryl Plus sutures were associated with significantly fewer SSIs and shorter hospital stays compared to traditional Monocryl. Although the overall complication, reoperation, and readmission rates showed nonsignificant trends favouring Monocryl Plus, the most notable benefits appeared in hypospadias repair, suggesting that suture choice might influence outcomes in this subgroup.

## 1. Introduction

Surgical site infections (SSIs) are infections that occur within 30 days after surgery, or within one year if the patient has implants, and involve either the incision site or deeper tissues at the surgical area. Despite progress in surgical techniques and infection prevention, SSIs continue to be a major cause of illness and death, placing a heavy burden on healthcare systems [1]. SSIs are among the most costly healthcare-associated infections, often resulting in extended hospital stays and readmissions. Additionally, the 30-day readmission rate, frequently caused by these infections, is used as a key indicator of quality of care [2,3].

In pediatric reconstructive urology, even minor infectious complications can delay recovery, lead to reoperation, or compromise functional and cosmetic outcomes, especially after delicate procedures like hypospadias repair, ureteral reimplantation, and pyeloplasty. Risk factors for SSIs include both patient-related and procedural factors. Patient-related elements involve non-modifiable characteristics such as sex and age, as well as modifiable aspects like overall health and nutritional status. Procedural factors encompass preoperative antisepsis, surgical hand hygiene, antibiotic prophylaxis, the duration of surgery, wound classification, and the type of suture material used [4,5,6].

The suture material itself can contribute to postoperative infection because sutures may act as a foreign body and surface for bacterial adherence and biofilm formation. This has led to the development of antimicrobial-coated sutures as an additional preventive strategy [7]. Triclosan-coated sutures, such as Monocryl Plus^®^ and PDS Plus^®^, are intended to inhibit bacterial colonization by releasing triclosan, a broad-spectrum antimicrobial that disrupts bacterial membrane synthesis. This coating provides localized antibacterial activity for up to 30 days after implantation without affecting tensile strength or causing tissue irritation [8].

Several randomized trials and meta-analyses have shown that triclosan-coated sutures significantly lower SSI rates across various surgical fields, including abdominal and colorectal surgery [9,10,11]. However, large multicenter studies, like the PROUD trial, did not verify a notable benefit, suggesting that the impact may vary based on wound class, surgical area, and patient population [12]. Evidence in urology and pediatric reconstructive surgery remains limited and somewhat contradictory. Some research indicates reduced SSI rates and fewer urethrocutaneous fistulas in hypospadias repair with triclosan-coated sutures, while other studies report no significant difference [13,14].

Monocryl^®^ and Monocryl Plus^®^ are monofilament sutures made from the polymer poly-p-dioxanone. Their smooth surface reduces tissue resistance and bacterial adhesion, and they cause minimal tissue reaction. As synthetic absorbable sutures, they hydrolyze into smaller monomers that are metabolized in the tissue. Monocryl Plus is coated with triclosan, providing extra antimicrobial protection during the early postoperative period [15,16].

While the benefit of triclosan-coated sutures has been explored in mixed and adult populations, pediatric urological reconstructive surgery remains underrepresented, particularly in large, procedure-specific cohorts with long-term follow-up. The aim of this study was to compare postoperative outcomes between traditional Monocryl^®^ and triclosan-coated Monocryl Plus^®^ sutures in pediatric patients undergoing reconstructive urological procedures, primarily focusing on surgical site infections, and secondarily on overall complications, reoperations, readmissions, and hospital stays.

## 2. Methods

### 2.1. Patients

**Figure 1:**
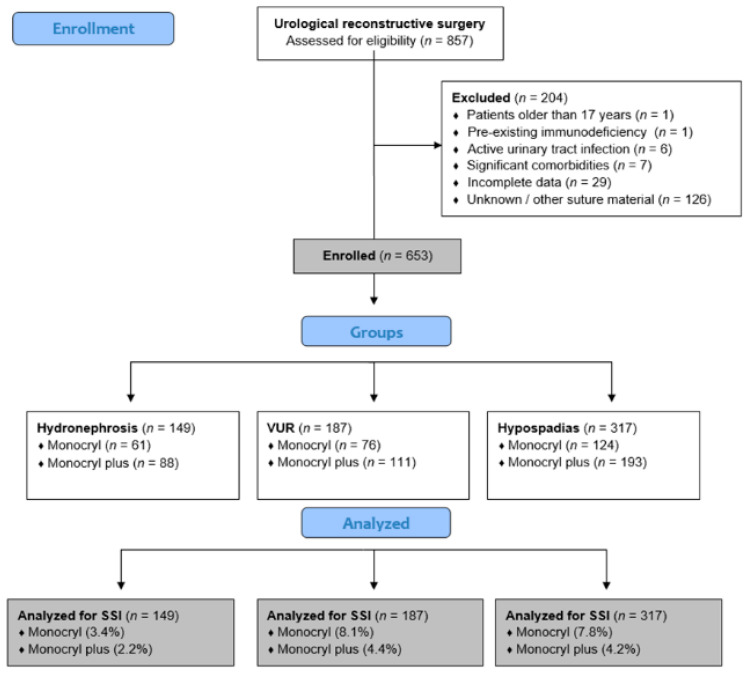
Flow-chart of the study.

A retrospective review of medical records for all pediatric patients who underwent urological reconstructive surgery at the Department of Pediatric Surgery, University Hospital of Split, from January 1, 2008, to December 31, 2025, was performed. From this group, patients treated for hydronephrosis, vesicoureteral reflux (VUR), or hypospadias were selected. After applying inclusion and exclusion criteria, 653 patients remained for analysis: 149 with hydronephrosis, 187 with VUR, and 317 with hypospadias. Each diagnostic group was divided into two suture groups, based on the suture material recorded in the operative documentation. In the first group, poliglecaprone 25 (Monocryl^®^, 4–6/0, Ethicon, Inc., a Johnson & Johnson company, Somerville, NJ, USA) was used, whereas in the second group triclosan-coated poliglecaprone 25 (Monocryl Plus^®^, 4–6/0, Ethicon, Inc., a Johnson & Johnson company, Somerville, NJ, USA) was applied. The assignment was non-random, based on surgeon preference and material availability at the time of surgery. All patients under 18 years old at surgery were eligible if they had complete intraoperative and postoperative documentation and at least twelve months of follow-up. Patients were excluded if they had pre-existing immunodeficiency, an active urinary tract infection at the time of surgery, or incomplete clinical records preventing accurate outcome assessment. The study flowchart is shown in Figure 1.

### 2.2. Ethical Aspects

This study was conducted in accordance with the ethical standards of the institutional research committee and the 1964 Declaration of Helsinki, along with its later amendments. The Institutional Review Board approved the protocol prior to data collection (approval number: 520-03/25-01/240; approval date: 30 October 2025). All patient data were anonymized before analysis to ensure patient confidentiality. Since this is a retrospective observational study, informed consent was waived or considered unnecessary according to institutional policy.

### 2.3. Outcomes of the Study

The main outcome of this study was the occurrence of surgical site infection (SSI) within the first 30 days after surgery, confirmed through standardized clinical criteria within 30 days postoperatively and, when clinically indicated, supported by microbiological culture results; however, microbiological confirmation was not uniformly available due to the retrospective nature of the study. Secondary outcomes included the overall rate of postoperative complications, reoperations, readmission rates, and the length of hospital stay measured in days from the initial procedure to discharge. Early and late complications were also documented and compared between groups.

### 2.4. Data Collection and Study Design

Data were retrospectively collected from both electronic and paper-based medical records. Demographic details included age at surgery, sex, height, weight, ASA classification, comorbidities, and body mass index (BMI) at the time of the procedure. Operative details such as the type and duration of surgery and the suture material used were systematically documented. Patients were assigned to the Monocryl^®^ or Monocryl Plus^®^ groups based on the intraoperative suture type recorded. Baseline characteristics were compared between groups to ensure the study population was homogeneous. Postoperative complications were divided into two categories. Early complications were defined as adverse events occurring within 30 days of surgery, such as surgical site infection, wound dehiscence, urinary leakage, catheter malfunction, or unplanned readmission. Late complications were those developing more than 30 days post-surgery, including anastomotic stricture or obstruction following pyeloplasty, recurrent or persistent vesicoureteral reflux, ureteral obstruction after reimplantation, or urethrocutaneous fistula and meatal stenosis after hypospadias repair. Additionally, the length of hospital stay, measured in days from the index procedure until discharge, was recorded for all patients. A prolonged hospital stay was defined as postoperative hospitalization exceeding 7 days and was analyzed both as a continuous variable (median, IQR) and as a dichotomous variable (>7 days) in regression models. Throughout the entire study period, SSI was defined according to standardized clinical criteria applied consistently in our institution. SSI surveillance was limited to the first 30 postoperative days and was based on inpatient records and scheduled postoperative outpatient visits, ensuring uniform outcome assessment across the study period. Overall complications refer to the number of patients experiencing one or more postoperative complications during the observation period (each patient counted once). Early and late complications are reported as event counts; some patients experienced both early and late events and may appear in both categories.

### 2.5. Surgical Techniques and Suturing Material

Surgical procedures were standardized based on diagnosis. In cases of hydronephrosis, Anderson–Hynes pyeloplasty was performed [17]. For VUR cases, ureteroneocystostomy [18,19] or the Cohen procedure [19] was used as the standard approach. In hypospadias repair, techniques such as TIP (tubularized incised plate) [20], MEMO (meatal mobilization) [21], MAGPI (meatoplasty and glanuloplasty) [22], or two-stage repairs [23] were employed depending on severity and the surgeon’s judgement. The suture material used was either Monocryl^®^ (uncoated poliglecaprone 25, Ethicon, Inc., a Johnson & Johnson company, Somerville, NJ, USA) or Monocryl Plus^®^ (triclosan-coated poliglecaprone 25, Ethicon, Inc., a Johnson & Johnson company, Somerville, NJ, USA). Both are absorbable monofilament sutures with similar mechanical and handling properties; the main difference is the antibacterial triclosan coating on Monocryl Plus^®^. Across all procedures, Monocryl^®^ and Monocryl Plus^®^ sutures were used in comparable anatomical locations, including urethral reconstruction, subcutaneous tissue, and skin closure. Suture size typically ranged from 4/0 to 6/0, selected according to patient age, tissue characteristics, and surgical site. Importantly, the choice of suture material was independent of the surgical technique used and was determined by surgeon preference and material availability rather than procedure complexity or severity.

### 2.6. Postoperative Protocol and Follow-Up

All patients received standardized preoperative antibiotic prophylaxis according to institutional protocol, consisting of a single intravenous dose of a first- or third-generation cephalosporin administered within 30–60 min before skin incision (or an appropriate alternative in case of beta-lactam allergy). At the end of surgery, standard wound dressings were applied. Postoperative antibiotic prophylaxis was administered according to institutional protocol until the removal of the catheter. In most cases, this involved a single daily dose of intravenous gentamicin or a third-generation cephalosporin, given for the duration of catheterization. Patients with active urinary tract infection at the time of surgery were excluded from the study. In patients considered at increased risk for postoperative infection (e.g., preoperative catheterization, previous failed surgery, or complex reconstruction), the same standardized perioperative antibiotic regimen was applied without additional or prolonged prophylaxis. Urinary catheters or stents were kept for a period determined by the surgeon (commonly 7–14 days in hypospadias, variable in other procedures). Discharge criteria were standardized and included no fever, stable vital signs, adequate oral intake, satisfactory wound condition, and removal of the urinary catheter or stent as required by the procedure. Patients were followed at scheduled intervals of 1, 3, 6, and 12 months after surgery and then annually when needed to monitor wound healing, identify late complications, and assess functional outcomes. Unplanned visits or interventions were also recorded. Postoperative follow-up schedules and wound assessment protocols remained unchanged throughout the study period.

### 2.7. Statistical Analysis

All analyses were conducted using IBM SPSS Statistics for Windows, Version 26.0 (IBM Corp., Armonk, NY, USA). Graphical plots were generated with Python version 3.10 (Python Software Foundation) using the Matplotlib library (version 3.8). Categorical variables were reported as frequencies and percentages, and group comparisons employed the chi-square test or Fisher’s exact test when expected cell counts were low. Continuous variables were summarized as mean ± standard deviation (SD) if normally distributed, or as median with interquartile range (IQR) if not. The Shapiro–Wilk test checked for normality. Independent samples *t*-tests or Mann–Whitney U tests were used for group comparisons as appropriate. Multivariate logistic regression analyzed the independent association between suture type (Monocryl vs. Monocryl Plus) and each outcome (surgical site infection, overall complications, reoperation, fistula formation, length of hospital stay), adjusting for relevant covariates, including age at surgery, operative duration, ASA classification, comorbidities, and diagnostic group. All models additionally included diagnostic group (hydronephrosis, vesicoureteral reflux, hypospadias) as a categorical covariate. Covariates included in the multivariate models were selected a priori based on established risk factors for surgical site infection in pediatric surgery and included age at surgery, operative duration, ASA classification, comorbidities, and diagnostic group. A two-sided *p*-value < 0.05 was considered statistically significant.

## 3. Results

### 3.1. Patient Characteristics

A total of 653 pediatric patients were included in the analysis: 149 with hydronephrosis, 187 with VUR, and 317 with hypospadias. Overall, Monocryl Plus^®^ sutures were used in 392 patients (60.0%), while Monocryl^®^ sutures were used in 261 patients (40.0%). The median age at surgery was 5 years (IQR 3–8), with no significant difference between the suture groups. Baseline demographic and perioperative characteristics, including sex distribution, height, weight, BMI, and operative duration, were comparable between groups. In both groups, nearly all patients received standard postoperative antibiotic prophylaxis until catheter or stent removal, with no significant difference in distribution between groups (Monocryl 97.7% vs. Monocryl Plus 98.2%, *p* = 0.72). The duration of prophylaxis, typically 7–14 days in hypospadias repairs and variable in other procedures, did not differ significantly across groups. The median follow-up was 36 months (IQR 24–60), with no differences between groups (*p* = 0.74). The proportion of procedures requiring intraoperative ureteral stenting or surgical drain placement did not differ significantly between suture groups. Baseline demographic and operative characteristics of patients according to suture type are shown in Table 1.

### 3.2. Postoperative Outcomes

**Figure 2:**
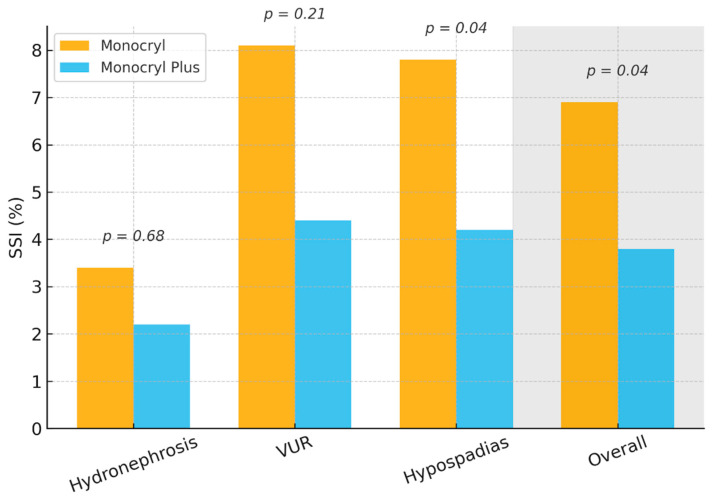
Surgical site infection (SSI) rates by diagnosis and suture type. VUR = vesicoureteral reflux.

The incidence of surgical site infection within the 30 days after surgery (primary outcome) was significantly lower in the Monocryl Plus group compared to the Monocryl group (3.8% vs. 6.9%, *p* = 0.04). Figure 2 shows SSI rates stratified by diagnosis and suture type, with consistently lower rates in the Monocryl Plus group and a statistically significant difference observed in hypospadias repair.

**Figure 3:**
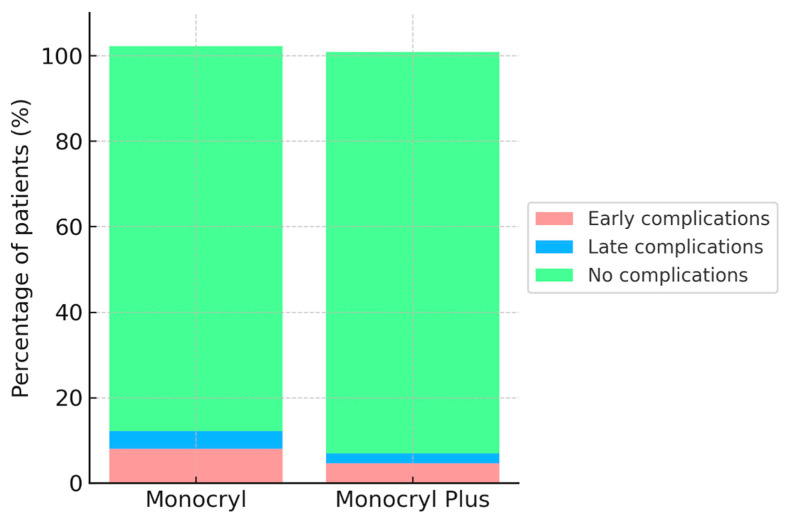
Distribution of postoperative complications in Monocryl and Monocryl Plus groups.

Regarding secondary outcomes, overall complication rates were lower in the Monocryl Plus group (6.1% vs. 10.0%), although the difference did not reach statistical significance (*p* = 0.07) (Figure 3).

**Figure 4:**
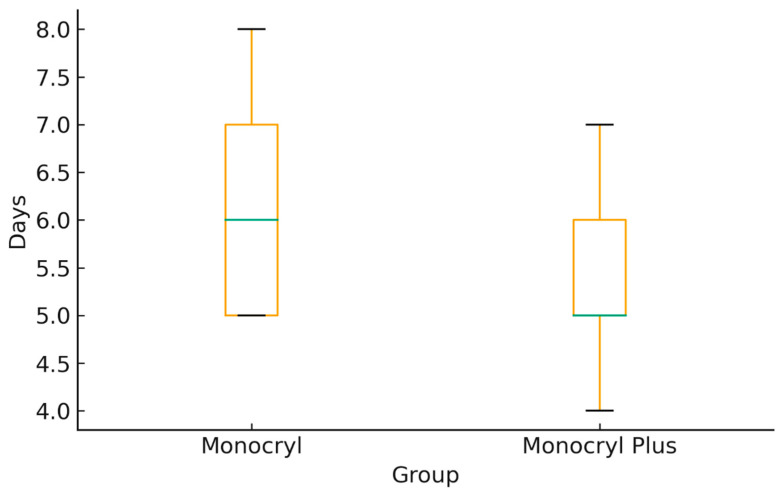
Length of hospital stay according to suture type.

The median length of hospital stay was shorter among patients treated with Monocryl Plus (5 days, IQR 4–7) compared with those treated with Monocryl (6 days, IQR 5–8; *p* = 0.02). Figure 4 illustrates the distribution of hospital length of stay, demonstrating a lower median and narrower interquartile range in the Monocryl Plus group.

Overall complications mainly included urinary tract infections, wound dehiscence, urinary leakage, and sepsis during the early postoperative period, with anastomotic strictures or recurrent reflux occurring in the late follow-up. Early complications were seen in 4.6% of Monocryl Plus patients and 8.0% of Monocryl patients (*p* = 0.06), while late complications appeared in 2.3% versus 4.2%, respectively (*p* = 0.18). Reoperation was needed in 1.8% of the Monocryl Plus group and 3.4% of the Monocryl group (*p* = 0.19). Likewise, hospital readmissions were somewhat lower in the Monocryl Plus group (2.6% vs. 5.0%, *p* = 0.12), though these differences did not reach statistical significance (Table 2).

Overall complications represent the number of patients experiencing at least one postoperative complication. Early and late complications are reported as separate events, and patients with both early and late events are represented in both categories; therefore, the sum of early and late events may exceed the number of overall complications.

### 3.3. Subgroup Analyses

When stratified by diagnosis, outcomes varied according to suture type (Table 3).

Other complications included meatal stenosis, wound dehiscence, urethral stricture, and bleeding; categories are not exclusive. Fistula formation is reported separately in the hypospadias subgroup. The distribution of hypospadias repair techniques (TIP, MAGPI, MEMO, and staged repairs) was comparable between the Monocryl^®^ and Monocryl Plus^®^ groups, reducing the likelihood that differences in outcomes were driven solely by technique selection.

#### 3.3.1. Hydronephrosis (*n* = 149)

Among 149 patients undergoing pyeloplasty, the incidence of surgical site infection was 3.4% in the Monocryl group and 2.2% in the Monocryl Plus group (*p* = 0.68). Overall complications occurred in 5.1% and 3.3%, respectively (*p* = 0.67). Reoperation was needed in 1.7% versus 1.1% (*p* = 0.78). The median hospital stay was 6 days (IQR 5–7) in both groups (*p* = 0.95).

#### 3.3.2. Vesicoureteral Reflux (*n* = 187)

Among 187 patients with VUR, surgical site infections occurred in 8.1% of Monocryl cases and 4.4% of Monocryl Plus cases (*p* = 0.21). Overall complication rates were 10.8% versus 5.3% (*p* = 0.18). Reoperation was needed in 4.1% versus 1.8% (*p* = 0.29). The median length of stay was 6 days (IQR 5–8) for Monocryl compared to 5 days (IQR 4–7) for Monocryl Plus (*p* = 0.04).

#### 3.3.3. Hypospadias (*n* = 317)

In 317 hypospadias repairs, surgical site infection occurred in 7.8% of Monocryl cases compared to 4.2% of Monocryl Plus cases (*p* = 0.04). Urethrocutaneous fistula formation was more common in the Monocryl group (12.2% vs. 6.5%, *p* = 0.03). Other complications occurred in 5.5% versus 3.2% (*p* = 0.19). Reoperation was needed in 3.9% versus 2.1% (*p* = 0.22). Readmission was recorded in 5.5% versus 2.6% (*p* = 0.17). The median hospital stay was 6 days (IQR 5–8) for Monocryl and 5 days (IQR 4–7) for Monocryl Plus (*p* = 0.01).

### 3.4. Multivariate Analysis

Multivariate logistic regression was conducted to determine whether the type of suture material independently affected postoperative outcomes, while adjusting for age, operative duration, ASA classification, and comorbidities. Use of Monocryl Plus was independently linked to a significantly lower risk of surgical site infection (OR 0.55, 95% CI 0.30–0.98, *p* = 0.04) and prolonged hospital stay beyond 7 days (OR 0.59, 95% CI 0.38–0.91, *p* = 0.02). A trend toward lower overall complication rates was also observed (OR 0.61, 95% CI 0.36–1.03), although it did not reach statistical significance (*p* = 0.06). No independent association was found between suture type and risk of reoperation (OR 0.52, 95% CI 0.20–1.28, *p* = 0.15) or hospital readmission (OR 0.68, 95% CI 0.30–1.52, *p* = 0.34) (Table 4).

Multivariate logistic regression adjusted for age at surgery, operative duration, ASA classification, and presence of comorbidities. Values < 1.0 indicate reduced odds of the outcome in the Monocryl Plus group compared with the Monocryl group.

## 4. Discussion

Surgical site infection (SSI) remains a relevant postoperative complication in pediatric urological reconstructive surgery, although reported rates are generally low, particularly in clean and clean-contaminated procedures [11,24,25]. Even low-grade wound infections can lead to prolonged hospitalization, additional interventions, and increased healthcare costs. Antibacterial-coated sutures were developed to reduce bacterial adherence and biofilm formation on suture material; however, their clinical benefit in pediatric populations has not been clearly established.

Evidence from adult and mixed-population studies indicates heterogeneous effects across different surgical procedures and patient groups, while pediatric-specific data remain limited and inconsistent [11,24,26,27].

In the present study, the overall incidence of SSI was low, reflecting contemporary standards of pediatric surgical care, as reported in randomized pediatric trials and large systematic reviews [11,25]. When procedures were analyzed collectively, the use of Monocryl Plus^®^ sutures was associated with a significantly lower overall SSI rate compared with standard Monocryl^®^. This finding suggests that, across a heterogeneous range of pediatric urological reconstructive operations, antibacterial suture coating may confer a measurable protective effect against postoperative wound infection, even in settings with low baseline infection risk. This observation aligns with findings from meta-analyses conducted across multiple surgical specialties [11,24,26,27,28].

Subgroup analyses demonstrated that this effect reached statistical significance only in hypospadias repair. In contrast, in hydronephrosis and vesicoureteral reflux surgery, SSI rates were numerically lower in the Monocryl Plus^®^ group but did not reach statistical significance. The lack of statistical significance in these subgroups is most likely attributable to intrinsically low baseline SSI rates and limited subgroup sample sizes, rather than the absence of a biological effect, a limitation frequently highlighted in pediatric SSI studies and randomized trials [25,27]. Hypospadias repair, however, involves longer operative times, extensive tissue dissection, urethral reconstruction, and postoperative urinary diversion, factors that may increase wound vulnerability, creating operative conditions in which the antibacterial properties of triclosan-coated sutures may have greater clinical relevance. Accordingly, the observed procedure-specific effect should be interpreted as related to operative features rather than as contradictory.

Hypospadias repair represents a distinct clinical entity within pediatric urology and is associated with specific risk factors for postoperative wound complications [21,29]. Previous pediatric studies comparing different absorbable suture materials in hypospadias repair have shown that suture-related factors can influence postoperative outcomes, including wound healing and complication rates [29]. Moreover, evidence from a large single-centre pediatric cohort demonstrated that triclosan-coated polydioxanone sutures were safe and associated with reduced SSI following hypospadias repair, further supporting the biological plausibility of a procedure-related benefit in this setting [13].

The present findings are also consistent with broader evidence indicating that the effectiveness of triclosan-coated sutures is influenced by baseline infection risk and operative complexity. Large systematic reviews and meta-analyses have demonstrated an overall reduction in SSI with triclosan-coated sutures across various surgical specialties, although heterogeneity between procedures, wound classes, and patient populations remains substantial [11,24,26,27,28,30]. Importantly, randomized pediatric data suggest that the benefit of antibacterial-coated sutures may be more pronounced in operations associated with increased wound vulnerability [25].

Triclosan-coated sutures have demonstrated reduced bacterial adherence and colonization at the suture–tissue interface, with a potential effect on early biofilm formation [3]. While such laboratory findings cannot be directly translated into clinical outcomes, they provide a plausible biological explanation for the observed reduction in SSI, particularly in surgically complex procedures characterized by prolonged exposure and tissue reconstruction, such as hypospadias repair.

Conversely, procedures such as pyeloplasty or ureteral reimplantation for vesicoureteral reflux are generally performed in cleaner operative fields, with limited tissue dissection and inherently low SSI risk. In these clinical circumstances, the incremental benefit of antibacterial suture coating may be difficult to detect, particularly in subgroup analyses with limited statistical power. Similar observations have been reported in other pediatric surgical contexts with low baseline SSI rates, where differences between suture materials did not consistently translate into statistically significant outcomes [31,32].

In addition to infection-related outcomes, the use of Monocryl Plus^®^ in the present study was associated with a shorter length of hospital stay. Length of stay is a multifactorial outcome influenced by institutional protocols, postoperative management, and social factors; nevertheless, reduced SSI rates and improved wound healing may reasonably contribute to earlier discharge. This observation is clinically relevant, as shorter hospitalization may reduce healthcare costs and patient burden without compromising patient safety.

The multifactorial nature of SSI must be emphasized. Surgical technique, tissue handling, tissue perfusion, suture line tension, urinary drainage, and postoperative wound care remain critical determinants of wound healing and infection prevention [33,34,35,36]. Antibacterial suture coating should therefore be regarded as an adjunct rather than a substitute for meticulous surgical practice. The absence of statistically significant differences in some procedural subgroups should not be interpreted as evidence of inefficacy, but rather as a reflection of low event rates and limited statistical power, a recurring limitation in pediatric SSI research [11,25].

Several limitations of this study merit consideration. Given the retrospective design, clear cause-and-effect relationships cannot be confirmed, and the findings may partly reflect factors not included in the analysis. The non-randomized allocation of suture material and the gradual adoption of triclosan-coated sutures over time may have introduced temporal confounding, as improvements in surgical technique, perioperative care, or institutional protocols may have occurred in parallel. Although baseline characteristics were comparable and multivariate adjustment was performed, residual confounding related to unmeasured time-dependent factors cannot be fully excluded. Although the overall cohort was relatively large, subgroup sample sizes were smaller, reducing the ability to detect modest differences between groups. In addition, the included procedures represent a heterogeneous group of pediatric urological reconstructions, and particularly in hypospadias repair, variations in surgical technique may contribute to outcome differences that cannot be fully separated from the effect of suture material in a retrospective design. Because the baseline SSI rate was low, several analyses were limited by small event numbers, resulting in borderline *p*-values and wide confidence intervals. Subgroup findings, particularly in smaller diagnostic groups, should therefore be interpreted as exploratory and hypothesis-generating rather than definitive. Microbiological data were not consistently available, precluding pathogen-specific analyses. In addition, several intraoperative factors known to influence SSI risk, such as wound class, skin antisepsis agent, glove change practices, and intraoperative field contamination, were not consistently documented in the retrospective records and could therefore not be included in the regression models. Nevertheless, the single-centre design, standardized surgical techniques, and consistent perioperative protocols enhance internal validity and support meaningful interpretation of procedure-level effects.

Taken together, these findings indicate that the clinical impact of triclosan-coated sutures in pediatric urological reconstruction varies according to surgical procedure and operative characteristics. While a significant reduction in overall SSI rates was observed when procedures were analyzed collectively, the benefit was most evident in hypospadias repair, a procedure associated with greater wound vulnerability. The magnitude of effect appears to depend on baseline infection risk and operative complexity rather than representing a uniform benefit across all pediatric urological operations.

## 5. Conclusions

In this single-centre retrospective study, the use of Monocryl Plus^®^ sutures was associated with a lower overall rate of surgical site infection in pediatric urological reconstructive surgery. In subgroup analyses, a statistically significant reduction in SSI was observed in hypospadias repair, whereas in hydronephrosis and vesicoureteral reflux surgery infection rates were numerically lower but did not reach statistical significance. An association between the use of antibacterial-coated sutures and a shorter length of hospital stay was also observed. Overall, these findings reflect procedure-specific associations rather than causal effects and indicate that the relationship between suture type and postoperative outcomes may vary according to procedure type, baseline infection risk, and operative complexity. Further prospective and randomized studies are required to determine whether these associations represent true causal effects.

## Figures and Tables

**Table 1:** Baseline demographic and operative characteristics of patients according to suture type.

Characteristic	Monocryl (*n* = 261)	Monocryl Plus (*n* = 392)	*p*
Age at surgery (years), median (IQR)	5 (3–8)	5 (3–8)	0.62 †
Male sex, *n* (%)	198 (75.9%)	296 (75.5%)	0.91 §
Height (cm), mean ± SD	114.2 ± 22.5	115.0 ± 23.1	0.78 ‡
Weight (kg), median (IQR)	22 (17–29)	22 (18–30)	0.67 †
BMI (kg/m^2^), mean ± SD	16.4 ± 2.7	16.6 ± 2.9	0.58 ‡
ASA I, *n* (%)	198 (75.9%)	296 (75.5%)	0.92 §
ASA II, *n* (%)	51 (19.5%)	75 (19.1%)	0.89 §
ASA III, *n* (%)	12 (4.6%)	21 (5.4%)	0.71¶
Any comorbidity, *n* (%)	36 (13.8%)	52 (13.3%)	0.89 §
Cardiac anomalies	9 (3.4%)	13 (3.3%)	0.94 ¶
Respiratory disease	11 (4.2%)	16 (4.1%)	0.97 §
Endocrine/metabolic disorder	7 (2.7%)	11 (2.8%)	0.96 ¶
Antibiotic prophylaxis, *n* (%)	255 (97.7%)	385 (98.2%)	0.72 §
Operative time (min), mean ± SD	92 ± 35	91 ± 34	0.84 ‡

ASA = American Society of Anesthesiologists physical status classification; BMI = body mass index; IQR = interquartile range; SD = standard deviation. † Mann–Whitney U test; ‡ Student’s *t*-test; § Chi-square test; ¶ Fisher’s exact test.

**Table 2:** Overall postoperative outcomes according to suture type.

Outcome	Monocryl (*n* = 261)	Monocryl Plus (*n* = 392)	*p*
Surgical site infection, *n* (%)	18 (6.9%)	15 (3.8%)	0.04 §
Overall complications, *n* (%)	26 (10.0%)	24 (6.1%)	0.07 §
Early complications (<30 days), *n* (%)	21 (8.0%)	18 (4.6%)	0.06 §
Late complications (>30 days), *n* (%)	11 (4.2%)	9 (2.3%)	0.18 §
Reoperation, *n* (%)	9 (3.4%)	7 (1.8%)	0.19 ¶
Readmission, *n* (%)	13 (5.0%)	10 (2.6%)	0.12 §
Length of stay (days), median (IQR)	6 (5–8)	5 (4–7)	0.02 †

Overall complications represent the number of patients with at least one postoperative complication (each patient counted once). Early and late complications are reported as events and may overlap in the same patient. IQR = interquartile range. † Mann–Whitney U test; § Chi-square test; ¶ Fisher’s exact test.

**Table 3:** Subgroup analyses of postoperative outcomes by diagnosis and suture type.

Outcome	Hydronephrosis (*n* = 149)		*p*	VUR (*n* = 187)		*p*	Hypospadias (*n* = 317)		*p*
	Monocryl (*n* = 59)	Monocryl Plus (*n* = 90)		Monocryl (*n* = 74)	Monocryl Plus (*n* = 113)		Monocryl (*n* = 128)	Monocryl Plus (*n* = 189)	
SSI, *n* (%)	2 (3.4%)	2 (2.2%)	0.68 ¶	6 (8.1%)	5 (4.4%)	0.21 §	10 (7.8%)	8 (4.2%)	0.04 §
Overall complications, *n* (%)	3 (5.1%)	3 (3.3%)	0.67 ¶	8 (10.8%)	6 (5.3%)	0.18 §	15 (11.7%)	15 (7.9%)	0.12 §
Early complications (<30 d), *n* (%)	2 (3.4%)	2 (2.2%)	0.68 ¶	6 (8.1%)	5 (4.4%)	0.21 §	13 (10.2%)	11 (5.8%)	0.09 §
Late complications (>30 d), *n* (%)	1 (1.7%)	1 (1.1%)	0.78 ¶	3 (4.1%)	2 (1.8%)	0.29 ¶	7 (5.5%)	6 (3.2%)	0.19 §
Reoperation, *n* (%)	1 (1.7%)	1 (1.1%)	0.78 ¶	3 (4.1%)	2 (1.8%)	0.29 ¶	5 (3.9%)	4 (2.1%)	0.22 ¶
Readmission, *n* (%)	2 (3.4%)	2 (2.2%)	0.68 ¶	4 (5.4%)	3 (2.7%)	0.31 §	7 (5.5%)	5 (2.6%)	0.17 §
LOS, median (IQR)	6 (5–7)	6 (5–7)	0.95 †	6 (5–8)	5 (4–7)	0.04 †	6 (5–8)	5 (4–7)	0.01 †

Procedure-specific complications are reported within the corresponding diagnostic subgroup. SSI = surgical site infection; VUR = vesicoureteral reflux; IQR = interquartile range; LOS = length of hospital stay. † Mann–Whitney U test; § Chi-square test; ¶ Fisher’s exact test.

**Table 4:** Multivariate logistic regression analysis of the association between suture type (Monocryl Plus vs. Monocryl) and postoperative outcomes.

Outcome	OR (95% CI)	*p*
Surgical site infection (SSI)	0.55 (0.30–0.98)	0.04
Overall complications	0.61 (0.36–1.03)	0.06
Reoperation	0.52 (0.20–1.28)	0.15
Readmission	0.68 (0.30–1.52)	0.34
Prolonged hospital stay (>7 days)	0.59 (0.38–0.91)	0.02

OR = odds ratio; CI = confidence interval; SSI = surgical site infection.

## Data Availability

The original contributions presented in this study are included in the article. Further inquiries can be directed to the corresponding author.

## References

[1] Calderwood M.S., Anderson D.J., Bratzler D.W., Dellinger E.P., Garcia-Houchins S., Maragakis L.L., Nyquist A.C., Perkins K.M., Preas M.A., Saiman L. (2023). Strategies to prevent surgical site infections in acute-care hospitals: 2022 Update. Infect. Control Hosp. Epidemiol..

[2] Petrosyan Y., Thavorn K., Maclure M., Smith G., McIsaac D.I., Schramm D., Moloo H., Preston R., Forster A.J. (2021). Long-term health outcomes and health system costs associated with surgical site infections: A retrospective cohort study. Ann. Surg..

[3] Jukić M., Antišić J., Pogorelić Z. (2023). Incidence and causes of 30-day readmission rate from discharge as an indicator of quality care in pediatric surgery. Acta Chir. Belg..

[4] Ban K.A., Minei J.P., Laronga C., Harbrecht B.G., Jensen E.H., Fry D.E., Itani K.M., Dellinger E.P., Ko C.Y., Duane T.M. (2017). American College of Surgeons and Surgical Infection Society: Surgical Site Infection Guidelines, 2016 Update. J. Am. Coll. Surg..

[5] Allegranzi B., Bischoff P., de Jonge S., Kubilay N.Z., Zayed B., Gomes S.M., Abbas M., Atema J.J., Gans S., van Rijen M. (2016). New WHO recommendations on preoperative measures for surgical site infection prevention: An evidence-based global perspective. Lancet Infect. Dis..

[6] Edmiston C.E., Lavin P., Spencer M., Borlaug G., Seabrook G.R., Leaper D. (2020). Antiseptic efficacy of an innovative perioperative surgical skin preparation: A confirmatory FDA phase 3 analysis. Infect. Control Hosp. Epidemiol..

[7] Alverdy J.C., Hyman N., Gilbert J. (2020). Re-examining causes of surgical site infections following elective surgery in the era of asepsis. Lancet Infect. Dis..

[8] Galam P., Desai Y., Patel A., Bajpayee L., Kumar Shetty K. (2025). Efficacy and Safety of MITSU AB™ Triclosan-Coated Sutures in Preventing Surgical Site Infections. Cureus.

[9] Otto-Lambertz C., Decker L., Adams A., Yagdiran A., Eysel P. (2023). Can triclosan-coated sutures reduce the postoperative rate of wound infection? Data from a systematic review and meta-analysis. Surgery.

[10] Miyoshi N., Fujino S., Clinical Study Group of Osaka University, Colorectal Cancer Treatment Group (CSGOCG) (2023). Triclosan-coated sutures to reduce surgical site infection in abdominal gastrointestinal surgery: A meta-analysis and systematic review. Surg. Open Sci..

[11] Jalalzadeh H., Timmer A.S., Buis D.R., Dreissen Y.E.M., Goosen J.H.M., Graveland H., Griekspoor M., IJpma F.F.A., van der Laan M.J., Schaad R.R. (2025). Triclosan-Containing Sutures for the Prevention of Surgical Site Infection: A Systematic Review and Meta-Analysis. JAMA Netw. Open..

[12] Bustamante Montalvo M., Cainzos M., Prieto Carreiras L., Castiñeira Piñeiro A., García Iglesias A., Fernandez Novo A., González Gómez L.M., Flores A., Diz Gil R., Fernández Baltar C. (2021). Evaluation of the effect of triclosan coated sutures in the prevention of surgical site infections in a Spanish hospital setting: A prospective, observational study. Infect. Prev. Pract..

[13] Pogorelić Z., Stričević L., Elezović Baloević S., Todorić J., Budimir D. (2024). Safety and Effectiveness of Triclosan-Coated Polydioxanone (PDS Plus) versus Uncoated Polydioxanone (PDS II) Sutures for Prevention of Surgical Site Infection after Hypospadias Repair in Children: A 10-Year Single Center Experience with 550 Hypospadias. Biomedicines.

[14] Borkar N., Tiwari C., Mohanty D., Baruah T.D., Mohanty M., Sinha C.K. (2024). Post-urethroplasty complications in hypospadias repair: A systematic review and meta-analysis comparing polydioxanone and polyglactin sutures. World J. Pediatr. Surg..

[15] Adkins J.M., Ahmar R.A., Yu H.D., Musick S.T., Alberico A.M. (2022). Comparison of Antimicrobial Activity Between Bacitracin-Soaked Sutures and Triclosan Coated Suture. J. Surg. Res..

[16] Baracs J., Huszár O., Sajjadi S.G., Horváth O.P. (2011). Surgical site infections after abdominal closure in colorectal surgery using triclosan-coated absorbable suture (PDS Plus) vs. uncoated sutures (PDS II): A randomized multicenter study. Surg. Infect..

[17] Pogorelić Z. (2022). Surgical approach to the treatment of urinary tract anomalies. Liječ. Vjesn..

[18] Todorić J., Budimir D., Saraga M., Košuljandić Đ., Arapović A., Šušnjar T., Todorić D., Furlan D., Milunović K.P., Meštrović J. (2014). Vesicoureteral reflux: Etiology, classification and diagnostic investigation. Pediatr. Croat..

[19] Fujii T., Satoh H., Sato A., Ishizuka Y., Izawa M., Morimoto Y., Shimono R. (2025). Lich-Gregoir vs. Cohen ureteral re-implantation surgery for bilateral vesicoureteral reflux: A propensity score analysis. Pediatr. Int..

[20] Jonardi P.A., Situmorang G.R., Wahyudi I., Rodjani A., Abbas T., Raharja P.A.R. (2025). Assessing Prognostic Studies of Tubularized Incised Plate Urethroplasty in Hypospadias: A Systematic Review of Methodological Rigor. Int. J. Urol..

[21] Pogorelić Z., Milovac B., Čohadžić T., Todorić J. (2024). Safety and effectiveness of meatal mobilization (MEMO) technique for glandular, coronal, and subcoronal hypospadias repair in children: A 5-year single-center study with 105 hypospadias. Biomedicines.

[22] Kızılöz H., Okçelik S., Temel M.C. (2021). MAGPI under local anaesthesia without catheter as an alternative to standard TIP procedure in distal hypospadias repair. Andrologia.

[23] Oktar T., Selvi I., Dönmez M.İ., Aydın B., Böyük A., Ziylan O. (2025). Two-stage repair for primary hypospadias: Functional and cosmetic outcomes in 145 cases with a follow-up period of over five years. J. Pediatr. Surg..

[24] Ahmed I., Boulton A.J., Rizvi S., Carlos W., Dickenson E., Smith N.A., Reed M. (2019). The use of triclosan-coated sutures to prevent surgical site infections: A systematic review and meta-analysis of the literature. BMJ Open.

[25] Renko M., Paalanne N., Tapiainen T., Hinkkainen M., Pokka T., Kinnula S., Sinikumpu J.-J., Uhari M., Serlo W. (2017). Triclosan-containing sutures versus ordinary sutures for reducing surgical site infections in children: A double-blind, randomised controlled trial. Lancet Infect. Dis..

[26] Edwards M., Graziadio S., Shore J., Schmitz N.D., Galvain T., Danker W.A., Kocaman M., Pournaras D.J., Bowley D.M., Hardy K.J. (2023). Plus sutures for preventing surgical site infection: A systematic review of clinical outcomes with economic and environmental models. BMC Surg..

[27] Apisarnthanarak A., Singh N., Bandong A.N., Madriaga G. (2015). Triclosan-coated sutures reduce the risk of surgical site infections: A systematic review and meta-analysis. Infect. Control Hosp. Epidemiol..

[28] Wang Z.X., Jiang C.P., Cao Y., Ding Y.T. (2013). Systematic review and meta-analysis of triclosan-coated sutures for the prevention of surgical-site infection. Br. J. Surg..

[29] Shirazi M., Haghpanah A., Dehghani A., Haghpanah S., Ghahartars M., Rahmanian M. (2022). Comparison of post-urethroplasty complication rates in pediatric cases with hypospadias using Vicryl or polydioxanone sutures. Asian J. Urol..

[30] Edmiston C.E., Seabrook G.R., Goheen M.P., Krepel C.J., Johnson C.P., Lewis B.D., Brown K.R., Towne J.B. (2006). Bacterial adherence to surgical sutures: Can antibacterial-coated sutures reduce the risk of microbial contamination?. J Am Coll Surg..

[31] Son T.N., Bao H.V. (2021). Long-term absorbable versus non-absorbable suture in laparoscopic percutaneous extraperitoneal closure of internal ring for inguinal hernia in children. J. Pediatr. Surg..

[32] Sykes A.G., Prieto J.M., Thangarajah H., Keller B.A., Kling K.M., Ignacio R.C., Lazar D.A. (2022). Modified laparoscopic gastrostomy tube placement in children: Does subcutaneous suture type matter?. J. Pediatr. Surg..

[33] McHugh S.M., Hill A.D., Humphreys H. (2011). Intraoperative technique as a factor in the prevention of surgical site infection. J. Hosp. Infect..

[34] Bath M.F., Davies J., Suresh R., Machesney M.R. (2022). Surgical site infections: A scoping review on current intraoperative prevention measures. Ann. R. Coll. Surg. Engl..

[35] Bucataru A., Balasoiu M., Ghenea A.E., Zlatian O.M., Vulcanescu D.D., Horhat F.G., Bagiu I.C., Sorop V.B., Sorop M.I., Oprisoni A. (2023). Factors Contributing to Surgical Site Infections: A Comprehensive Systematic Review of Etiology and Risk Factors. Clin. Pract..

[36] Prabhu R., Mohamed M.S., Alhammali T., Ghareb R., Doddamane Prasanna S., Abdelglil M., Soffar A., Elkohail A., Teama M., Khalil N. (2025). Modern Surgical Site Infection Prevention: Evidence, Gaps, and Future Directions. Cureus.

